# 1.E. Workshop: Accelerating the action on SDGs applying a One Health approach

**DOI:** 10.1093/eurpub/ckac129.016

**Published:** 2022-10-25

**Authors:** Organised by: Instituto Universitário Egas Moniz, NOVA National School of Public Health (Portugal), Chair persons: Ricardo Assunção (Portugal), Susana Viegas (Portugal)

## Abstract

In the sequence of a pandemic, with a tremendous impact in our lives, more than never the challenges for humankind are enormous. Despite the future is impossible to predict, there are some certain trends that could compromise the health of humans, living systems and Earth. Environmental degradation and climate change, poverty and inequalities, demography, shocks and crises, constitute some of the core dares that should be seriously considered. Recognizing the importance of tackling the challenges posed to humankind, world's governments signed an agreement to eradicate poverty, improve the living standards and well-being of all people, promote peace and more inclusive societies, and reverse the trend of environmental degradation, in a document called 2030 Agenda for Sustainable Development. This document contains the 17 Sustainable Development Goals (SDGs), promoting the development in a balanced way, leaving no one behind. After this historic initiative, several strategies, debates and efforts have been advanced, tried and implemented to tackle these challenges. However, sophisticated policy responses of preparedness, investment and cooperation are still needed. One Health is considered an approach to designing and implementing programmes, policies, legislation and research in which multiple sectors communicate and work together to achieve better public health outcomes. The current challenges identified, e.g. infectious diseases, climate change, the sustainability of food systems, the destruction of biodiversity, could affect transversally health, are related differently with several SDGs, and demand for a holistic approach that should be efficient enough to tackle these threats. Through presenting some case studies, this workshop aims to promote the discussion regarding the implementation of a One Health approach to accelerate the action targeting SDGs accomplishment, focusing particularly on actions that can have an impact on Health in a broader sense, i.e. human, animal and environment health. Each presentation will emphasize specific examples (air pollution, food systems and climate change, intensive animal production, biodiversity), relating the concept of One Health and the linked SDGs that could be faced through this approach. Altogether, these presentations will highlight the need for a multi- and trans-sectorial intervention, using the concept of One Health to maximize the co-benefits within the three domains, human, animal, and environmental health. The challenges are enormous, but as Nelson Mandela said, “It always seems impossible until it's done”.

**Key messages:**

• The importance of the One Health approach to tackle the goals on sustainable development, health and wellbeing.

• The One Health approach brings more easily co-benefits in different sectors that result in more relevant gains in Health in the three domains (human, animal and environment health).

